# 3D histopathology of human tumours by fast clearing and ultramicroscopy

**DOI:** 10.1038/s41598-020-71737-w

**Published:** 2020-10-19

**Authors:** Inna Sabdyusheva Litschauer, Klaus Becker, Saiedeh Saghafi, Simone Ballke, Christine Bollwein, Meraaj Foroughipour, Julia Gaugeler, Massih Foroughipour, Viktória Schavelová, Viktória László, Balazs Döme, Christine Brostjan, Wilko Weichert, Hans-Ulrich Dodt

**Affiliations:** 1 Department of Bioelectronics, TU Wien, Vienna, Austria; 2 Center for Brain Research, Medical University of Vienna, Vienna, Austria; 3 Department of Surgery, Anna Spiegel Center of Translational Research, Medical University of Vienna, Vienna, Austria; 4 Institute of Pathology, TUM School of Medicine, Technical University of Munich, Munich, Germany

**Keywords:** Optical imaging, 3-D reconstruction, Imaging, Software, Microscopy, Light-sheet microscopy, Cancer, Breast cancer, Cancer imaging, Tumour angiogenesis, Tumour heterogeneity, Biological techniques, Cancer, Health care, Oncology

## Abstract

Here, we describe a novel approach that allows pathologists to three-dimensionally analyse malignant tissues, including the tumour-host tissue interface. Our visualization technique utilizes a combination of ultrafast chemical tissue clearing and light-sheet microscopy to obtain virtual slices and 3D reconstructions of up to multiple centimetre sized tumour resectates. For the clearing of tumours we propose a preparation technique comprising three steps: (a) Fixation and enhancement of tissue autofluorescence with formalin/5-sulfosalicylic acid. (b) Ultrafast active chemical dehydration with 2,2-dimethoxypropane and (c) refractive index matching with dibenzyl ether at up to 56 °C. After clearing, the tumour resectates are imaged. The images are computationally post-processed for contrast enhancement and artefact removal and then 3D reconstructed. Importantly, the sequence a–c is fully reversible, allowing the morphological correlation of one and the same histological structures, once visualized with our novel technique and once visualized by standard H&E- and IHC-staining. After reverting the clearing procedure followed by standard H&E processing, the hallmarks of ductal carcinoma in situ (DCIS) found in the cleared samples could be successfully correlated with the corresponding structures present in H&E and IHC staining. Since the imaging of several thousands of optical sections is a fast process, it is possible to analyse a larger part of the tumour than by mechanical slicing. As this also adds further information about the 3D structure of malignancies, we expect that our technology will become a valuable addition for histological diagnosis in clinical pathology.

## Introduction

Breast cancer is the most common malignancy in women^1^. The largest histological subgroup of invasive breast cancers is formed by invasive breast cancer of no special type (NST), commonly known as ductal carcinoma. Ductal carcinoma in situ (DCIS) is regarded to be its pre-invasive lesion which is, in contrast to the invasive carcinoma, confined to the mammary ductal-lobular system due to a preserved myoepithelial layer. Despite the comprehensive improvement in the field of breast cancer biology, especially in molecular diagnostics and the expansion of targeted therapies, the primary treatment option of both entities is a locoregional tumour resection with the aim of tumour-free resection margins. The guideline recommendation of minimal margin width is currently set to 2 mm provided that post-operative radiation therapy is performed. This threshold is considered to be sufficient enough to control the risk of local recurrence^2–4^.

After the surgical resection, a histopathological examination of the anatomical specimen is performed according to standard procedures.

Modern pathology relies on a decades-proven framework of 2D histopathological analysis, where tissue resectates are routinely processed by formalin fixation and paraffin embedding of representative tissue samples (FFPE). The paraffin blocks obtained thereby are cut with a microtome and then placed onto glass slides for haematoxylin and eosin (H&E) and/or immunohistochemistry (IHC) staining. This whole procedure takes up to a few days before the images are obtained. In general, only one to two histological sections of a 3–4 mm thick tissue block are produced, apart from especially selected cases, e.g. biopsies or sentinel lymph nodes which are processed by several serial or step sections. Considering that a single section is approximately 5 µm thick, the undersampling factor is about 600–800 with this technique. Although there is a significant risk that important stage-determining hallmarks of cancer are missed, the FFPE technique is still regarded as the gold standard in histopathological tissue analysis. However, it is crucial for the pathologist to see most of the tumour in its immediate vicinity, especially its marginal areas.

In recent years, scientists worldwide tried to improve the situation by inventing novel histopathological preparation and imaging techniques^5,6^. To our knowledge, the only one among these approaches that is currently used in clinical practice is the whole slide imaging (WSI) technique, where entire glass-mounted H&E-stained slices are scanned. The resulting images can be analysed with specially designed software and represent a convenient tool for the modern histopathology^7^. The drawbacks of this approach are the same as for standard tissue preparation, i.e. artefacts that occur due to the mechanical slicing and staining. Additionally, the scanning time for a single slide is between 5–15 min with an average of 10 min. Usually, the slides are scanned in batches of 80 to 120 and the scanning-time per batch is 12–24 h^8^. The resulting file-size varies from 48 MB to several GB per slide, depending on resolution. These numbers vary depending on the model of scanner, as this branch of digital histopathology is constantly developing, providing novel, improved systems^9,10^.

Attempts to transfer this existing technique from 2 to 3D, i.e. to create 3D-reconstructions from the series of cut and stained single sections of FFPE-processed specimen gave some interesting results^11–14^. However, due to the irreversible nature of this method, tissue is completely used up and therefore no longer available for certain additional analyses like the increasingly applied immunohistochemical testing for PD-L1 in the future. In the context of PD-L1 testing unstained sections should not be stored longer than 2–3 months, otherwise there is the risk of false negative staining results, meaning that the patient could be deprived of a potentially highly effective anti-tumoural immunotherapy. Moreover, being extremely time- and labour-intensive it would hardly be applicable in clinical practice.

Another attempt made with multiphoton imaging of optically cleared archive-compatible FFPE tissue^15^ was limited by the size of the specimen that can be scanned. Also, immunostaining of lymphatic microvasculature and blood vessels in cleared bladder samples of a few mm size required several weeks^16^ and was limited to depict tumour vascularization.

Intraoperative pathology using fluorescence excitation would be a useful option, if it could be introduced in routine healthcare practice. However, it is presently limited to the surface scanning of freshly resected tissue^17^. Confocal microscopy^18^, as well as light sheet microscopy of needle-core biopsies^19–21^ or the specimen surface provides only information about a tiny part of the relatively large sample^6,22^.

**Figure 1:**
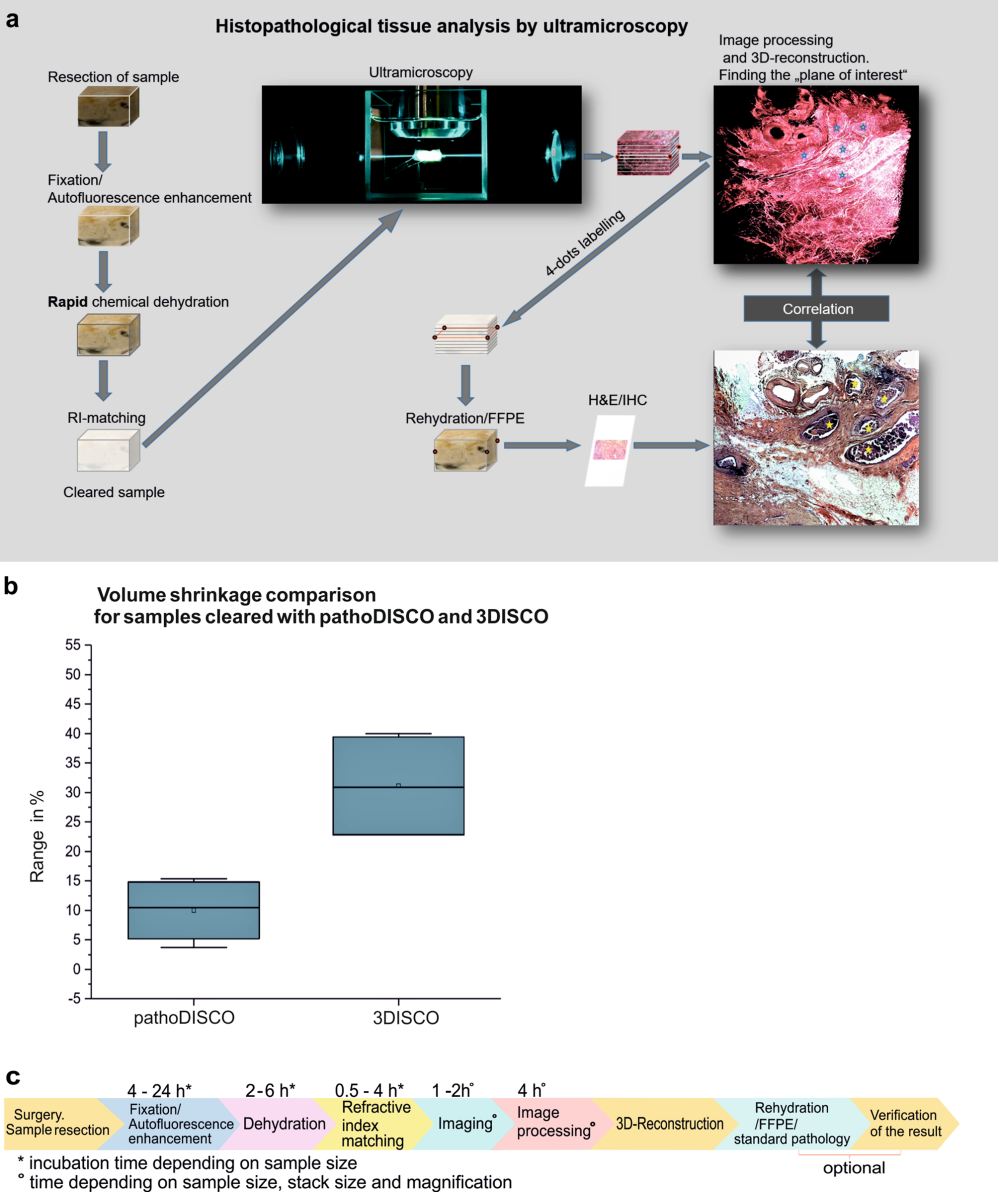
Tissue processing with pathoDISCO. (**a**) Workflow of reversible tissue clearing and 3D-imaging of the solid tumours. (**b**) Volume shrinkage comparison (in %) for samples, cleared with pathoDISCO and 3DISCO (n = 16). (**c**) Timeline of tissue processing from surgery to 3D-reconstruction.

In this study we present a novel tissue clearing and optical approach that has a high potential to provide a comprehensive three-dimensional overview of the tumour and its microenvironment. Due to its inexpensive, fast and easy performance it should be applicable for routine postoperative pathology in a not so far future (Fig. 1a).

## Results

### Ultrafast chemical clearing of tumour biopsies

For imaging by light sheet microscopy^23–25^ the tumour resectates have to be rendered transparent. To this purpose a number of novel chemical tissue clearing approaches were suggested in the recent years. One part of them relies on refractive index matching with lipophilic organic solvents as e.g. BABB (2 parts benzyl benzoate and 1 part benzyl alcohol)^26^ or dibenzyl ether (3DISCO)^27,28^, the other part on aqueous-solutions not requiring previous tissue dehydration, as e.g. CLARITY^29^ or CUBIC^30^.

We tested several of these clearing approaches with respect to their usability for routine cancer diagnostics. In all cases, we encountered major drawbacks, which made these approaches unsuitable for a potential routine application in clinical pathology. Either the clearing times were too long (weeks to months) or the method did not reliably and reproducibly clear large samples. We therefore developed a novel clearing approach (pathoDISCO) that is sufficiently fast to be applied in routine pathology. We found that our clearing technique renders resectates from breast tissue that are even larger than the size of a 30 × 20 × 5 mm standard histology cassette transparent in less than 48 h (Supplementary Fig. S1).

### Selective autofluorescence enhancement

Prior to dehydration and clearing we apply selective autofluorescence enhancement^31–36^ in order to highlight structures of interest in the cancer tissue. Although the standard fixative formalin is a potent autofluorescence enhancer^37^, we found that formalin fixation alone is not sufficient for an optimal visualization of tumour resectates. We found that the addition of 5% 5-sulfosalicylic acid dehydrate (2-Hydroxy-5-sulfobenzoic acid, PubChem CID: 2723734) to the 4% formalin fixative markedly boosts the autofluorescence of selected biological tissues. 5-Sulfosalicylic acid dehydrate, further mentioned here as 5ssA is widely used in forensics as a blood fixative, where it is typically applied before staining to fix the dye to the substrate^38^. We discovered that 5ssA has two effects on our cancer samples: on the one hand, it enhances the autofluorescence of particular tissue compounds; on the other hand it deeply acidifies the specimens, which is a prerequisite for the following chemical dehydration process. The 5ssA/formalin mixture can also be applied for reprocessing of formalin-fixed and paraffin embedded (FFPE) samples with our technique by washing off the paraffin from the FFPE-blocks with xylene (Merck, Germany) and ethanol, acidifying them with 5% 5ssA and then processing them the same way as fresh-formalin-fixed samples (Supplementary Fig. S2).

### Ultrafast chemical tissue dehydration

Passive tissue dehydration, e.g. with alcohol or tetrahydrofuran (THF)^39,40^, relies on a simple physical diffusion process where the water content of the sample is stepwise substituted by a dehydration medium. For this, several changes of the dehydration agent (usually 5–7) are required. After each change the samples have to be incubated until the concentration gradient between inside and outside of the sample is equilibrated. Unfortunately, the required diffusion times exponentially increase with the size of the sample, so that the totally required dehydration times for cm-sized tissue samples can easily become several weeks.

To speed up dehydration, we replaced the passive tissue dehydration by an active dehydration process utilizing 2,2-dimethoxypropane (DMP). DMP has been applied for dehydrating biological specimen before, mainly in the field of electron microscopy^41–43^, but—to our knowledge—was never used for dehydration as part of a clearing process of large biological tissue samples. For chemical dehydration, we incubate the samples that have previously been acidified with 5ssA, for 4–12 h in DMP, depending on the size of the sample. DMP chemically reacts with the water content of the tissue. The products of this reaction are methanol and acetone (1). The start of the reaction is well observable, as the medium becomes turbid and cools down rapidly, due to the endothermic (but yet spontaneous) nature of this process. The H^+^ ions provided by 5ssA initiate the reaction. The reaction products acetone and methanol are common tissue dehydration agents themselves. The equilibrium of this hydrolysis reaction is almost completely on the products side so that about 5.8 g (8 ml) of DMP can remove up to 1 g of water.

The amount of a chemically unreactive dehydration medium required for the same purpose would be more than 10 times higher^43^.1

With DMP the dehydration speed is only limited by its tissue penetration rate^43^. Since DMP is also a potent solvent for lipids^44^, it penetrates the cell membranes much faster than water. Noticeably, the grade of shrinkage we observed with DMP dehydration is much lower compared to passive dehydration, e.g. with tetrahydrofuran (THF), although DMP acts much faster than passive dehydration agents. The average volume shrinkage of breast cancer tissue resectates (N = 16) dehydrated with DMP is 10%, compared to 31% for samples dehydrated with THF (Fig. 1b, Supplementary material, Table S1) Even for several cm large samples the incubation times required with DMP would usually not exceed 6 h. In contrast to passive dehydration techniques, dehydration with DMP does not require more than one exchange of the medium, leading to less effort in time and a significant reduction of costs and wastage of reagents. The incubation times are not critical and may be prolonged for a better fitting into the respective workflow without any damage of the tissue (Fig. 1c).

### Refractive index matching

Following dehydration, the samples were immersed in DBE until they became transparent (usually 2–6 h at room temperature). We found that the exact clearing time depends on the type and cellular composition of the specimens. Loose connective tissue and adipose tissues become transparent much faster than dense connective tissues, e.g. skin or cartilage. We found out that by increasing the temperature to 56 °C the incubation time can be drastically shortened to less than one hour without any unwanted side-effects. An intermediate change of DBE after 30–60 (depending on the sample size) minutes is performed to remove residuals of methanol originating from the dehydration process. Following RI matching, the transparency of the tissue was quantified using an USAF- test chart (United States Air Force resolution test chart, MIL-STD-150A, a standard of 1951 from Edmund Optics, Germany) (Figs. 4a–c, 5a; Supplementary Fig. S1b,d, Fig. S2c, Fig. S3b, Table S2).

## Image acquisition

**Figure 2:**
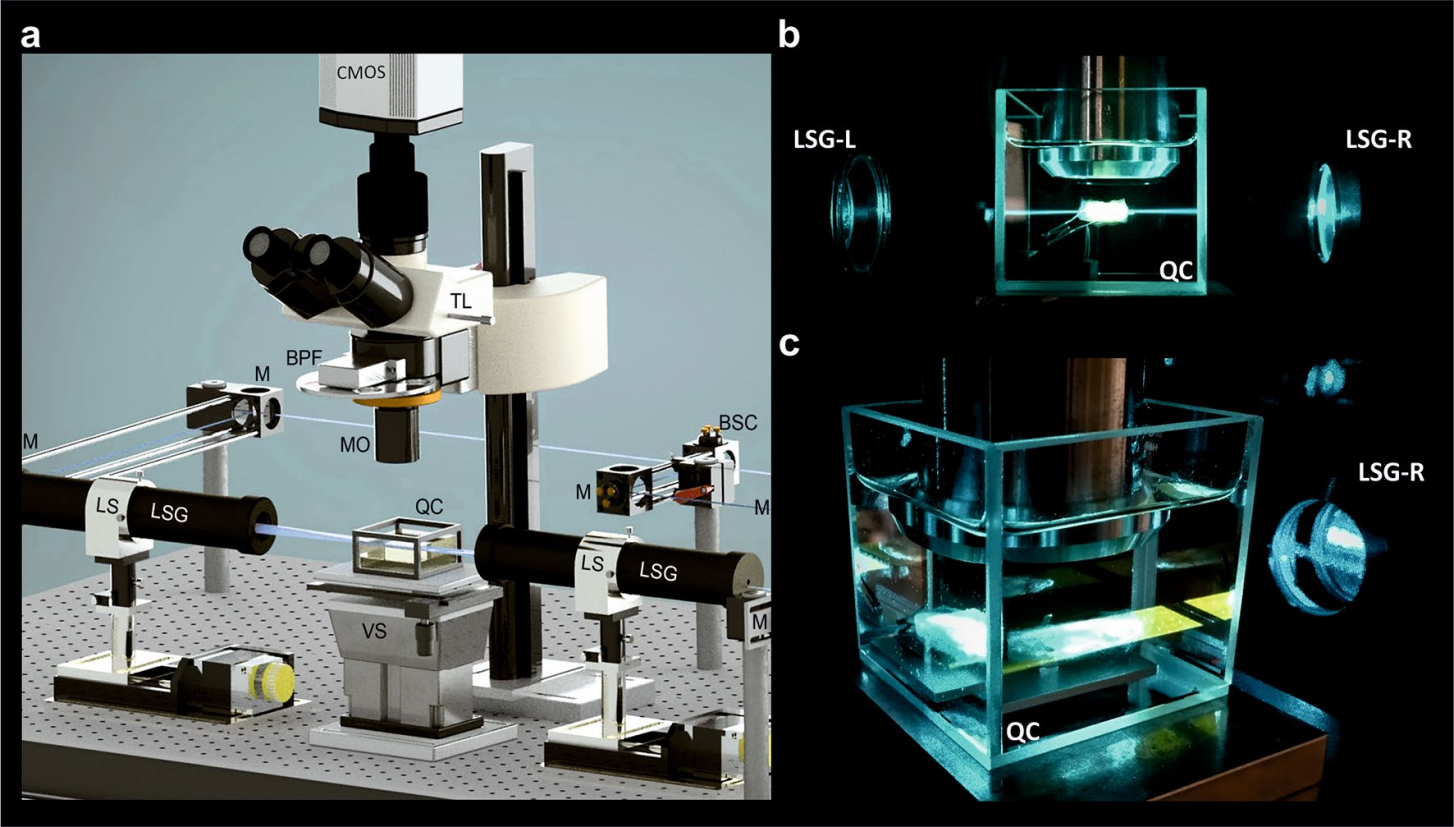
Ultramicroscopy setup. Recording of the large tissue sample. (**a**) Ultramicroscopy setup: Sapphire laser unit for fluorescence excitation (not shown), a beam splitter cube (BSC), 45 degrees silver mirrors (M), two light sheet generator units (LSG), two linear stages (LS) that move the LSG units along the beam propagation axis (z) for superimposing the center of the light sheet in the center of the biological sample, a computer controlled stage for moving the sample through the light sheet vertically (VS), a quartz container (QC), filled with imaging media (DBE). The detecting unit contains × 2, × 4 or × 16 objective equipped with a modulator (MO) for compensating refractive index mismatch, a tube lens (TL) equipped with a band pass filters (BPF) wheel, and a CMOS Camera. (**b**,**c**) Cancer sample recording with × 2 magnification.

The images based on selectively enhanced autofluorescence of the tumour resectates were acquired using a custom-made ultramicroscopy setup (Fig. 2) as described in^45^. We established that the optimal autofluorescence excitation was achieved by using a laser of 488 nm wavelength. The emitted fluorescence light was filtered by an optical band pass filter with cut-offs at 550 ± 49 nm.

**Figure 3:**
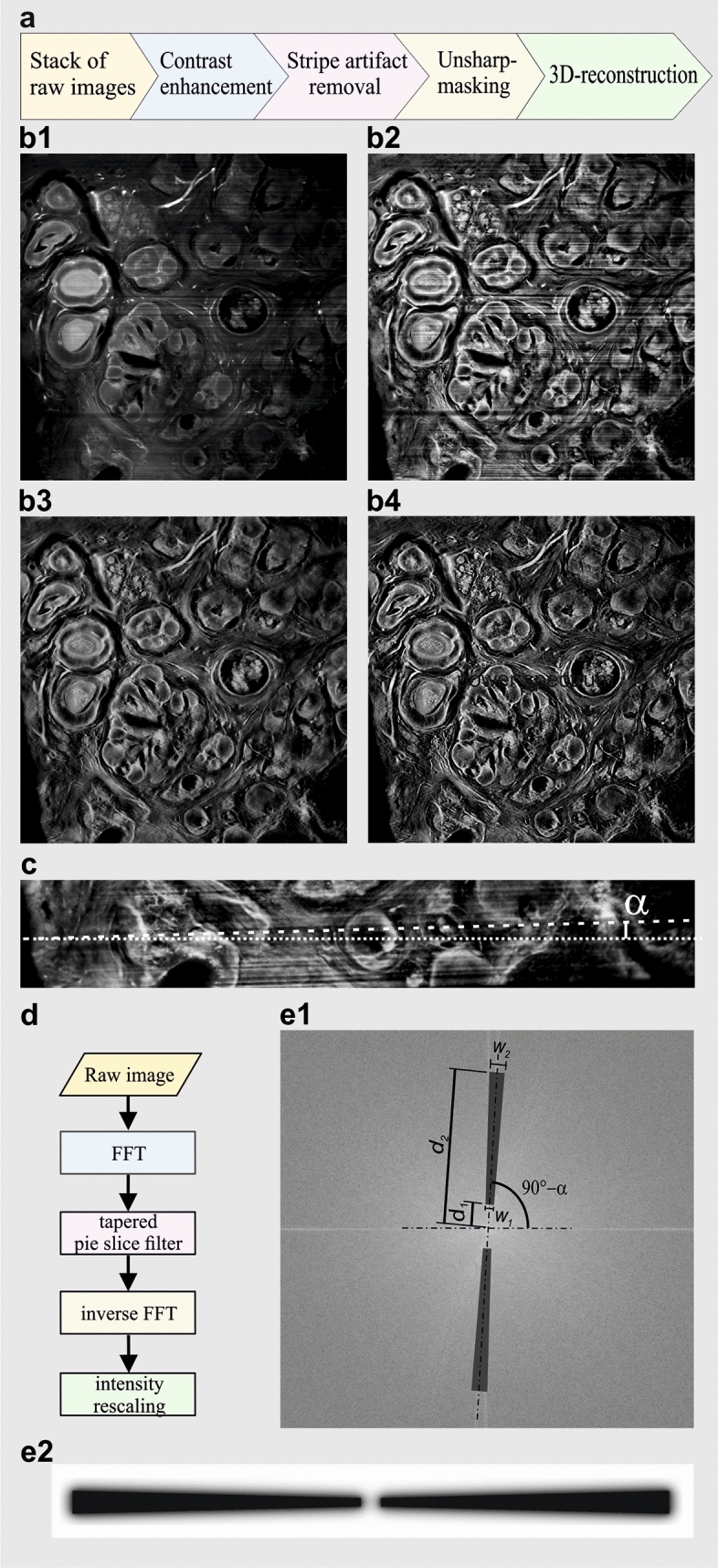
Computational improvement of cancer sample recordings. (**a**) Image processing chain for cancer biopsies. The UM image stacks are contrast enhanced by contrast limited histogram equilibration (CLAHE), followed by stripe artifact removal using a matched 2D Fourier transform slope filter and unsharp masking. (**b1**) Representative slice of an UM data set obtained from a breast cancer biopsy before post-processing. (**b2**) Same as in (**b1**) after contrast enhancement using CLAHE. The visibility of information encoded in small brightness differences is clearly enhanced. (**b3**) Stripe artifacts generated during UM recording have been removed via a matched 2D Fourier domain slope filter. (**b4**) Finally, the image is slightly sharpened via unsharp-masking to further enhance the visibility of fine details. (**c**) UM recordings often exhibit stripe shaped artefacts originating from light absorbing structures that are persistent to the clearing procedure. By obstructing the light sheet these structures produce visible shadows that can include an angle α with the horizontal image edges depending on the camera orientation. (**d**) To remove the stripe artefact the images are Fourier transformed and multiplied with a filter mask cutting out a pie-slice shaped piece of the spectrum matching the angular direction α of the stipes. After inverse transformation and rescaling a stripe suppressed image is obtained. (**e1**) Design of the pie shaped filter. The angular direction α of the stripes corresponds to an angle of 90-α in the 2D power spectrum. The angular direction and the shape of the pie slice filter can be optimized in the software by varying α and the distances *d*_1_, *d*_2_, *w*_1_, and *w*_2_. This allows to match bandwidth and direction sensitivity of the filter in order to find a parameter combination providing best possible stripe suppression at minimal costs of blurring artefacts or ringing. (**e2**) To reduce ringing artefacts due to a hard frequency cutoffs, the edges of the pie shaped filter exhibit a smooth Gaussian transition profile. All image processing steps were performed using custom-made software written in MATLAB (MathWorks, Germany) and Visual Basic.Net (Microsoft, USA). The programs can be obtained from klaus.becker@twien.ac.at upon reasonable request.

After recording, the acquired image stacks were subjected to contrast limited adaptive histogram equilibration (CLAHE)^46^, stripe artefact removal by directional spatial filtering in the frequency domain^47^, and unsharp-masking for final improvement of sharpness. We found that these processing steps, when applied in this sequence, markedly enhance contrast and perceptibility of fine details (Fig. 3a–e2). For achieving optimal results by CLAHE the number of grey levels in the images should be as high as possible (e.g. 16 bit corresponding to 65,535 grey levels), since the algorithm transforms unnoticeable small brightness differences into larger differences well perceivable for the human eye.

Since all applied image processing steps operate on 2D images they are not computationally cost intensive as e.g. 3D-deconvolution approaches, which require three-dimensional input data. Therefore, the entire post-processing chain can principally be performed in real time already during recording using a state-of-the-art multiprocessor computer.

### 3D reconstruction

**Figure 4:**
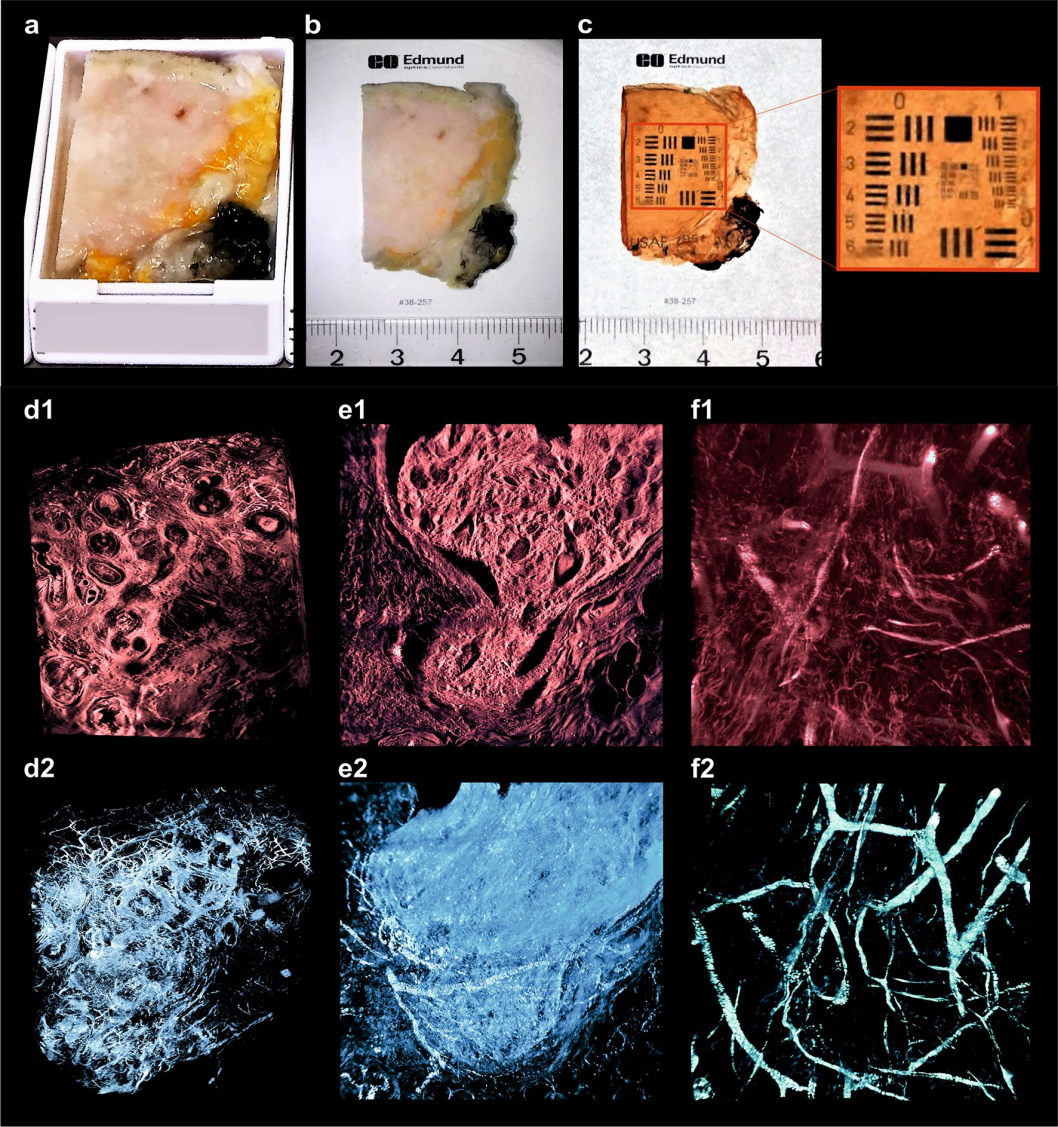
3D-histopathology applied to human breast neoplasms. Highlighting cancer-relevant tissue structures. (**a**,**b**) Uncleared breast tissue specimen. (**c**) Same specimen after chemical tissue clearing. (**d**–**f**) Representative images of 3D-reconstructions of specimen. (**d1**) Selected plane of DCIS sample recorded with × 2 magnification. (**d2**) Same 3D-reconstruction with highlighted blood vessels. (**e1**) Selected plane of 3D-reconstruction of breast carcinoma specimen recorded with × 16 magnification. (**e2**) Same 3D-reconstruction, with highlighted blood vessels and sites of mitotic activity. (**f1**) 3D reconstruction of breast carcinoma sample recorded with × 16 magnification. (**f2**) Separately visualized blood vessels of the same sample (see movies to Fig. 4 in Supplementary material).

**Figure 5:**
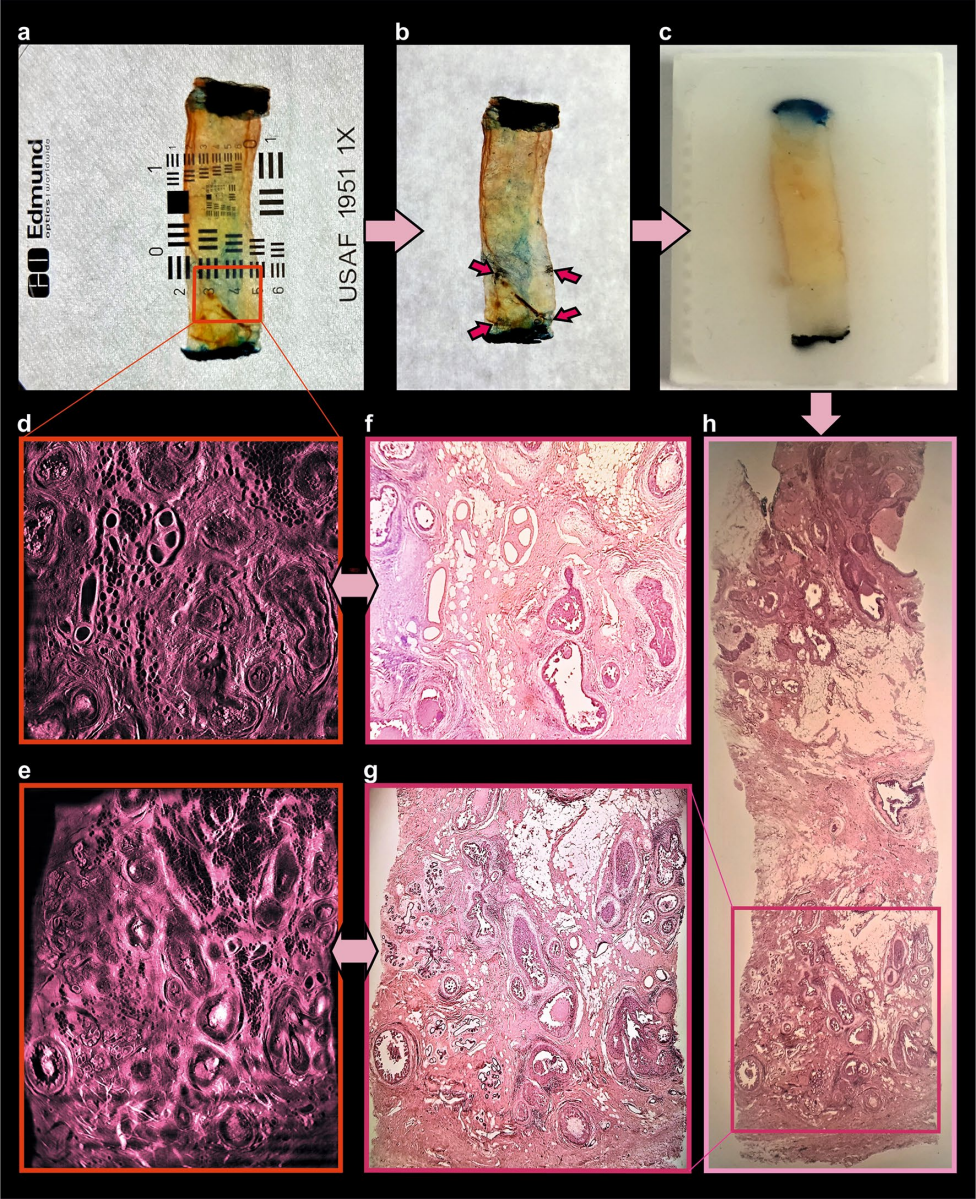
Post-processing of cleared specimen with standard histology methods. Comparison of obtained images. (**a**) Cleared specimen. (**b**) 4-dots labelling (red arrows) of the optical “area of interest”, found within the UM-recording. (**c**) Same sample, embedded in paraffin. (**d**,**e**) Optical sections of 3D-reconstruction of the specimen (see movie to Fig. 5d in Supplementary material), corresponding to (**f**,**g**) H&E-stained histological sections (× 2 magnification). (**h**) Whole-specimen cut with microtome and stained with H&E.

**Figure 6:**
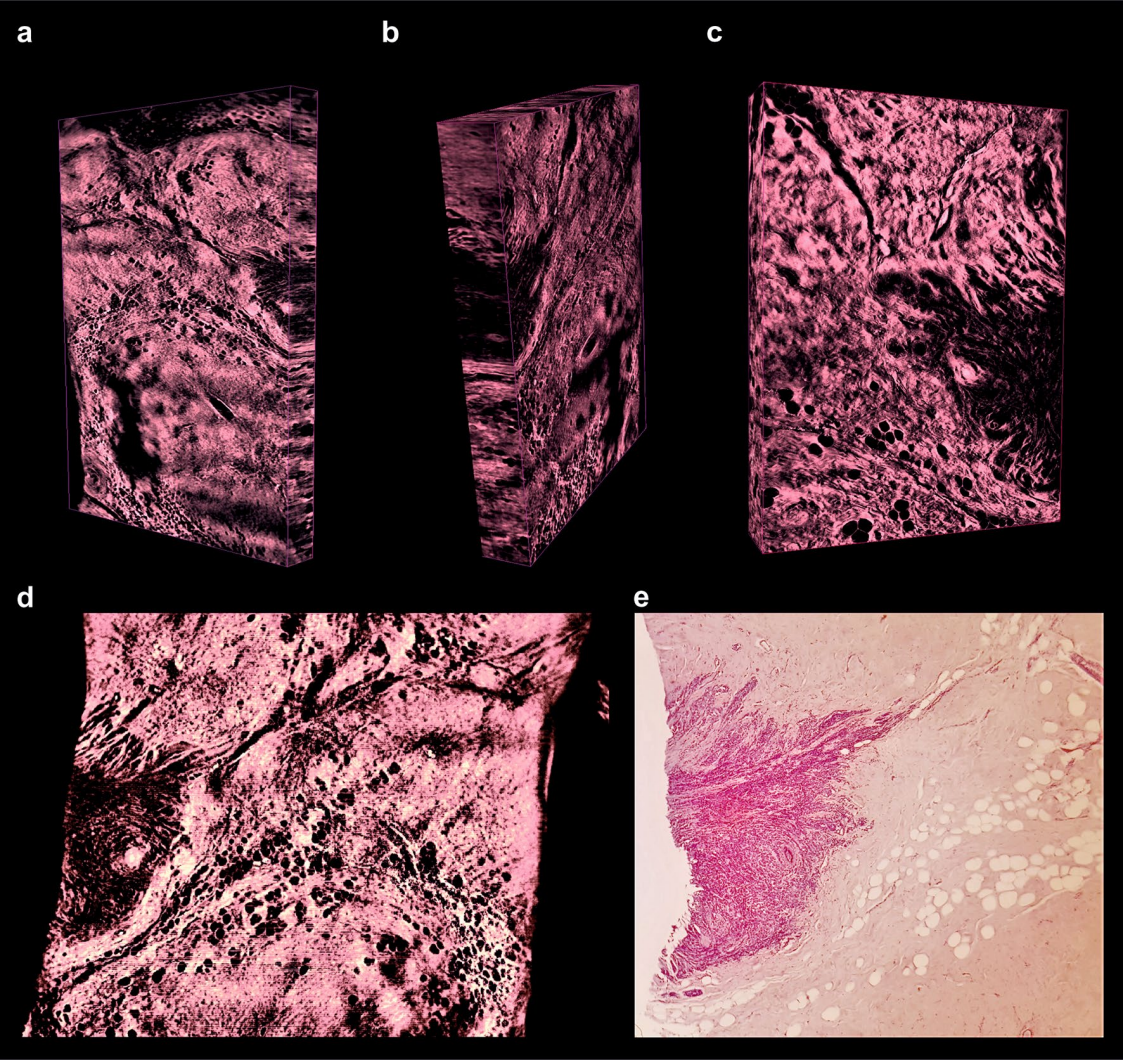
3D imaging of low-grade invasive lobular adenocarcinoma of breast. (**a–c**) Representative 3D reconstructions of the sample recorded with × 2 and × 4 magnification, captured from different perspectives; sites of high cellular density corresponding to the invasive carcinoma are visualized as dark areas. (**d**) Optical section of 3D reconstruction of the sample recorded with × 4 magnification. (**e**) Corresponding physical section of the specimen (post-clearing), processed with standard histology, stained with H&E and recorded with × 5 magnification (see movies to Fig. 6 in Supplementary material).

**Figure 7:**
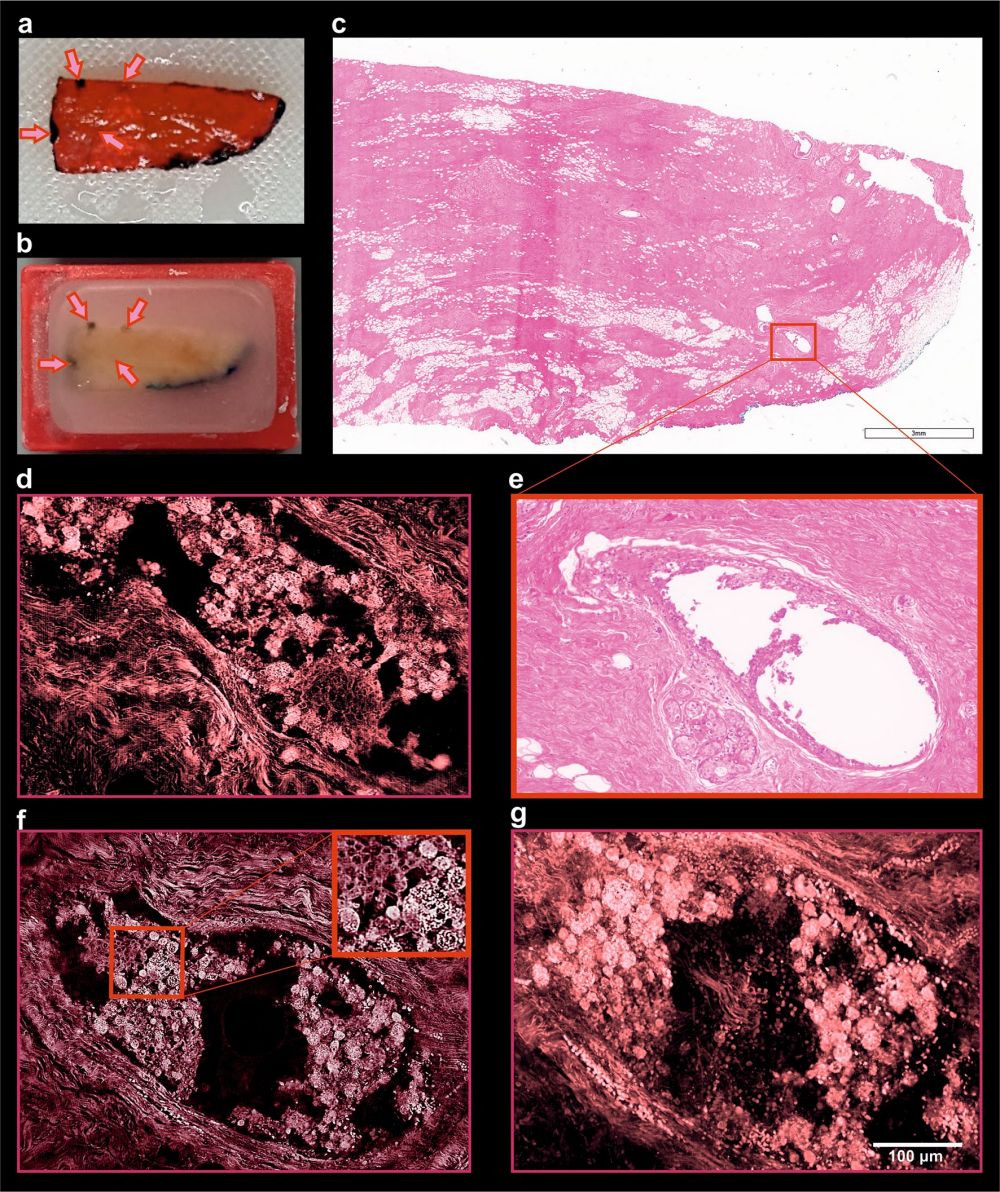
Comparison of tissue compounds preservation of specimen processed with pathoDISCO and standard histology. (**a**) Cleared sample labelled with histo-marker. (**b**) Post-clearing paraffin-embedded sample with 4-dots labelling. Arrows showing the sites of 4-dots labelling; (**c**) whole specimen slice, proceeded with standard FFPE/H&E; (**e**) zoom-in, showing the loss of intra-ductal cellular mass due to the mechanical tissue processing. (**d**,**f**,**g**) Several optical planes of the 3D reconstruction of the sample, recorded with UM at × 16 magnification (see movies to Fig. 7 in Supplementary material).

After pre-processing, stacks of 600–2,000 images were three-dimensionally reconstructed using the visualization software AMIRA 6.7 (Thermofisher, Germany). Since the tumour recordings are monochromatic, different types of tissue have to be distinguished by their characteristic autofluorescence intensity (Fig. 4d1,e1,f1). We found that the highest autofluorescence intensities correspond to erythrocytes and microcalcifications, vascular structures and collagen fibres. Consequently, vascular structures and collagen fibres become visible using a 2 × or 4 × magnification (Fig. 4d2,e2), erythrocytes (Figs. 4f2, 7g) can be recognized using a 16 × magnification. Nuclei (16 × magnification) (Fig.  7d,f) and the fat cells (2 × or 4 × magnification) (Figs. 5d,e, 6d) appear dark due to their low autofluorescence.

To ease up detection and recognition of relevant tissue structures by human vision, we developed several colour maps which translate the brightness differences into colour differences. We also performed an intensity-based threshold segmentation to highlight the diagnosis-relevant structures within the 3D-reconstructions. We could demonstrate that this allows visualizing some hallmarks of cancer.

### Reversibility of the clearing process

To be able to compare our method with standard histology, we developed a protocol that can reverse the clearing process to the fixed hydrated phase, so that the samples can be subjected to a secondary processing with the standard FFPE/H&E-technique. We demonstrated that the cleared samples can be stepwise rehydrated and FFPE/H&E processed as usual formalin fixed material (Fig. 5a–c). This is important, since it allows a direct comparison of the same morphological structures visualized with our technique (Figs. 5d,e, 6a–d, 7d,f,g) versus standard FFPE/H&E processing (Figs. 5f–h, 6e, 7c,e).

**Figure 8:**
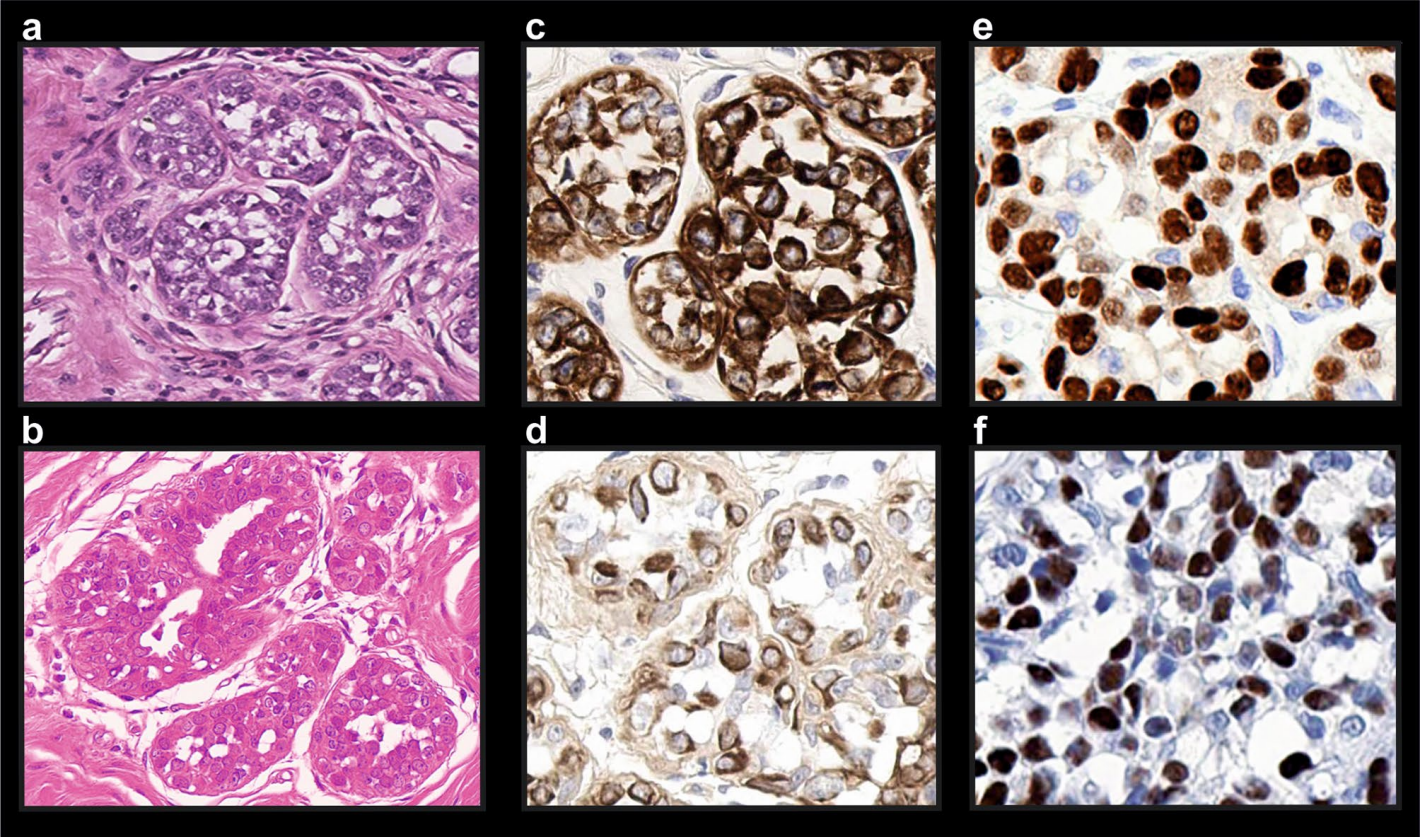
Comparison of post-UM and routinely processed FFPE-samples, acquired with × 40 magnification. (**a**) Sample, processed with standard histology, and stained with H&E; (**b**) post-UM sample, processed with FFPE and stained with H&E; (**c**) same sample, processed with standard histology and stained routinely with anti-cKpan (pan-keratin) and (**e**) anti-PR (progesterone receptor) antibodies; (**d**) Post-UM sample, processed with FFPE and stained with anti-cKpan (pan-keratin) and (**f**) anti-PR (progesterone receptor) antibodies at the pathology department.

To facilitate the relocation of previously 3D-imaged regions in the H&E stained slices obtained from the reprocessed samples, the approximate position of the imaging plane that had been used was marked with 4 dots at the sides of the specimen (Figs. 5b, 7a,b; Supplementary Fig. S3a–g). This labelling allowed us to identify the area that was previously imaged by light sheet microscopy (Supplementary Fig. S3e,h). The plane of slicing of the paraffin block could then be adjusted accordingly. In comparison to routinely performed FFPE/H&E samples, the histological images of post-UM samples were slightly more reddish, due to the acidification step with 5ssA (Figs. 6e, 8b).

The nuclei appear somewhat brighter, showing well-defined areas of condensed chromatin (nucleoli), the fine structural details however are preserved in the post-UM samples as well (Fig. 8a,b).

Moreover, we were able to depict the entirety of the pre-invasive intraductal proliferation of ductal carcinoma in situ using 16 × magnification objective. In comparison to the post-UM images obtained from the same specimen processed with standard histology (Fig. 7c,e), the intraductal cellular masses of the sample, processed with pathoDISCO remain well-preserved (Fig. 7d,f,g).

### Immunohistochemistry

In clinical pathology immunohistochemistry (IHC) is performed as an additional tool next to conventional H&E staining in order to correctly classify neoplasms or other disease conditions or to predict response to targeted therapies. Amongst others, IHC is applied to confirm the origin of tumours and to distinguish primary tumours from metastases, making it an essential diagnostic tool in cancer diagnosis^48^.

To test the compatibility of our clearing method with IHC staining protocols, we stained breast tissue samples that were previously cleared and recorded with UM with antibodies against pan-keratin and progesterone receptor. These two types of antibodies are commonly used for IHC staining in routine pathology, progesterone receptor especially in the field of gynecopathology. The samples stained after ultramicroscopy were slightly paler compared to reference samples that had not been cleared and rehydrated (Fig. 8c–f). But both, keratin and progesterone receptor expression were clearly detectable in previously cleared UM samples. This is not a drawback either, as evaluation of cytokeratin staining is solely based on its presence or absence and assessment of hormone receptor status is based on the percentage of stained tumour cell nuclei independent of staining intensity. Immunohistochemical assessment of Her2 status however is different as staining intensity and staining pattern are taken into consideration for the three-tiered classification system. Her2 status however has no therapeutic significance in DCIS and is therefore not routinely performed.

## Discussion

Obtaining a fast and precise diagnosis from surgically resected tissue is not only essential for the assessment of further therapy steps in the individual case but also for the development of improved cancer treatment strategies in the future. Standard histological processing of cancer resectates can be time-consuming and only a limited number of histological sections can be analysed in a reasonable timeframe. In order to improve this situation, we developed a technique that allows to clear, image and three-dimensionally reconstruct tumour resectates up to the size of a standard histological cassette. In contrast to previously published studies, where needle-core biopsies^21,49^, surface scanning or imaging of several mm-thick slices of cleared specimen were performed^6^, our method can be used for clearing of tissue samples in a size-range of several centimetres. A major advantage of our method is the possibility to reconstruct the whole tumour in its spatial vicinity, including the complete tumour margins in 3D, from up to several thousands of optical sections per sample.

Several hallmarks of cancer are common prognostic factors for the patients, since they provide additional information besides origin and type of the tumour. The concept of tumour microenvironment has gained increasing interest in recent years. An important component of this microenvironment constitutes neovascularization, not only as a reflection of tumour malignancy but also in terms of a potential therapeutic target^50^. Recently published studies described several novel methods for imaging vascular structures of human colon tissue^51^, as well as lymphatic vasculature of the human bladder^16^. The resulting images and 3D-reconstructions of the vascular structures provide an opportunity for studying tumour biology, but are not sufficiently resolved to allow diagnostics like vascular or lymphatic system invasion. Moreover, these techniques seem to be difficult to apply in a routine histopathology due to the absence of further cancer-hallmarks obtained by these reconstructions, as well as due to the complexity of the protocols involving antibody-staining.

Via selective autofluorescence enhancement, we were able to elucidate the vasculature in tumour resectates, as well as milk ducts, and cell aggregations within.

An interesting approach using the CUBIC clearing^52^, describes a clearing and imaging method, compatible with immunohistochemistry and standard histology after rehydration. Applied to specimen of up to 5 mm in size, it produced imaging results of anatomical structures in the lung (alveoli) and lymph-nodes (nodules). The approach relied mainly on the fluorescent staining of nuclei, so images similar to H&E staining could not be obtained. This loss of histological information and the processing time of over a week limits its clinical applicability.

Another noteworthy study published in 2018 used the CLARITY clearing for 3D-microscopy of human brain tissue blocks in the size of 3 × 3 × 5 mm, adapted for validation by magnetic resonance imaging (MRI)^53^. This approach allowed to depict several cell types in the human brain, like neurons, astrocytes and myelinated fibres. A major drawback of this approach is that it is extremely time-consuming. Including the antibody-staining part, the whole tissue processing takes from several weeks to 14 months (with an average of 10 months), making this approach unfeasible for clinical practice.

Recently, the possibility of combining the antibody-staining with both aqueous-based^53–55^ and solvent-based^56–58^ tissue clearing protocols were reported. Each of these described techniques represents a valuable approach for the research proposes, however, due to the long incubation times on the one hand, and moreover due to the necessity of large amounts of the expensive antibodies on the other hand- they seem to be not adequate in a routine clinical histopathology.

With pathoDISCO, the entire process including dehydration, refractive index matching, computational post-processing and 3D-imaging can be performed in 1–3 days. This lies within the scope of standard histological work-up. It thus fits well in the normal post-operative time-frame where the patient stays at the hospital. The reversibility of the pathoDISCO protocol allows performing an optional H&E- and IHC-staining of the same tissue regions that have been cleared and 3D reconstructed before.

This is a crucial advantage for further validating and optimizing the pathoDISCO protocol in clinical practice. After clearing and imaging it is possible to stain the corresponding tissue section of a certain “plane of interest” obtained by light sheet microscopy either with H&E or with an antibody of choice. This validation process may in future be supported by using pattern recognition software, which provide quantitative quality descriptors assessing the informational overlap between the H&E slice and the corresponding optical sections^59–61^. Our suggested clearing method is inexpensive and easy to perform so that it can be integrated into the workflow of any routine histology lab. As pathoDISCO can be performed within 1–3 days, pathologists can get the information of 3D-imaging simultaneously with standard histological sections. This means that there will not be any time delay, but even a gain of time if additional step sections are needed. At the beginning of a stepwise introduction of our technique into clinical praxis, pathoDISCO could be used solely for a screening of leftover tissues not needed for the standard pathology. In a second step it could be used for preliminary screening of gross sections, providing an overview of the whole specimen. Based on this overview, the pathologists can decide which part of the specimen should be additionally processed with the standard laboratory techniques.

We successfully applied our clearing technique to breast neoplasms. A limitation of our approach is attributable to the inter- and intra-tumoural heterogeneity, thus the optimal incubation times may vary within a certain range and should be individually adjusted. Regarding the preliminary results it seems to be straightforward to adapt the protocols to other types of tumour tissues. In general, specimens that are rich in loose connective or adipose tissue are faster to clear than tumour tissues of high cellular density.

We could show that our ultrafast tissue clearing technique can also be used for imaging of archived samples after deparaffinization of the tissue blocks. This ability opens the avenue to *post-hoc* analysis of the huge biobanks of archived tumour tissues around the world. Where the 3D structure of tumours is of relevance, pathoDISCO will provide completely new insights in tumour biology in the future.

## Materials and methods

### Ultrafast clearing of neoplasms

#### Obtaining of resectates

The tissue samples were provided by the Institute of Pathology of the TU Munich. Human tissue was used after written informed consent and approval from the Ethics Review Committee of the Technical University of Munich (77/17 S). Generally, we used the tissue material which remained after finalizing all the standard diagnostic procedures. The specimens were dissected, labelled and fixed, according to standard procedures. All methods described further were carried out in accordance with relevant guidelines.

#### Fixation

The samples were fixed immediately after resection in buffered 4% paraformaldehyde, further referred to as PFA or formalin (Carl Roth, Germany) for at least 12 h at room temperature (RT).

#### Selective autofluorescence enhancement

For selective autofluorescence enhancement, the PFA-fixed tissue samples were incubated in a solution consisting of 4% formalin and 5% 5-sulfosalicylic acid dehydrate (Merck, Germany), further referred to as 5ssA, in Dulbecco’s PBS without Ca^2+^ and Mg^2+^, (Thermo Fisher Scientific, Germany), at RT*.* The pH of the solution was 2.5. If possible, the freshly resected tissue can also be fixed directly in this mixture for at least 12 h, saving the first fixation step with PFA. After fixation the samples were dehydrated with 2,2-dimethoxypropane (Sigma Aldrich, Germany).

#### Rapid chemical dehydration

For chemical dehydration, the samples acidified with 5% 5ssA were incubated in 2,2-dimethoxypropane, further referred to as DMP (Sigma Aldrich, Germany) for 4–24 h, depending on their size, tissue type and consistency. The onset of the hydrolysis of DMP is directly observable, since the liquid becomes turbid and rapidly cools down. As a potent lipid solver^44^, DMP penetrates the cell membranes, causing the release of water-fat-residuals and some other debris into the medium. To remove this debris, the samples were briefly rinsed in 100% methanol (Merck, Germany) and further transferred into absolute methanol with a small amount of a molecular sieve of 30 nm mesh width (Honeywell Fluka, Germany). The samples were incubated on a shaker (60–80 rpm) for 6–24 h, depending on the size and cellular composition of the specimen, i.e. samples, consisting mainly of adipose or loose connective tissue required shorter incubation times than the samples consisting of collagen-rich dense connective tissue or solid tumour tissues of high cellular density. For each dehydration step the incubation times can be prolonged, depending on the schedule of the laboratory, without any damage to the tissue.

#### Refractive index matching

The dehydrated samples were incubated 2–6 h in dibenzyl ether, further referred to as DBE (Merck, Germany) for refractive index matching until they became transparent. For shortening the required incubation times, in some cases the temperature was increased up to 56 °C. After approximately 30 min the DBE was changed to remove residues of methanol originating from the dehydration procedure. After the samples are clear they can directly be imaged or be further kept in the DBE for storage. The transparency of the cleared samples was quantified by an USAF-test chart (MIL-STD-150A, Edmund Optics, Germany) (Supplementary material, Table S2).

#### Reprocessing of H&E stained paraffin blocks

To demonstrate that we can also work with archived material, we additionally cleared tissue derived from paraffin blocks that remained after the standard FFPE-based diagnostics. To this purpose a deparaffinization step was carried out in order to remove the paraffin from the samples. The paraffin was washed out by incubating the paraffin blocks in excessive amounts of xylene (Merck, Germany) on a shaker (60–80 rpm) at a room temperature for 12 h with one intermediate medium change. Rehydration of the deparaffinised tissue samples was attained by immersing them in a decreasing concentration series of ethanol: 100%, 80%, 60% of ethanol (Lactan, Austria) mixed with water and 40%, 20%, 5% of ethanol mixed in Dulbecco’s PBS without Ca^2+^ and Mg^2+^ (Thermo Fisher Scientific, Germany). All further processing steps were carried out in the same way as for the fresh or formalin-preserved tumour resectates.

### Image acquisition and computational post-processing

#### Ultramicroscopy

The tumour resectates were imaged using a custom-made ultramicroscope setup as described in Saghafi et al. (2013). For recording, the cleared samples were placed into glass cuvettes (Hellma, Germany) filled with DBE. For samples not exceeding a volume of approximately 1 cm^3^ a cuvette of 5 × 5 × 5 cm^3^ was used. A custom-made chamber of 9 × 11 × 6 cm^3^ was used for larger specimen. The sample was fixed in the centre of the chamber by a custom-made holder made of a metal vascular clamp (S&T, Germany) glued onto a custom-built 30′ ramp, made of polyoxymethylene (POM). Fluorescence excitation was done via a 488 nm Sapphire laser of maximally 500 mW power (Coherent, Germany). The images are acquired by the detection part of the UM comprising the objectives (2 ×, 4 ×), equipped with custom made modulators to compensate refractive index mismatch and an optical band pass filter (49 nm full bandwidth centred at ± 550 nm) followed by a scientific grade CMOS camera with 2,560 × 2,160 pixel resolution Andor Neo (Andor Technologies, Ireland).

For imaging with 16 × resolution we used a commercially available objective HC Fluotar (Leica Biosystems, Germany), with an adjustable RI-modulator, designed for imaging in clearing media. After passing an optical band pass filter with at 550 nm full bandwidth centred at ± 49 nm the emitted fluorescence light was recorded using an Andor Neo CMOS camera with 2,560 × 2,160 pixel resolution (Andor, Ireland).

#### Contrast limited adaptive histogram equalization (CLAHE)

The images were contrast enhanced using the CLAHE algorithm provided by the MATLAB Image Processing Toolbox (MathWorks, Germany). CLAHE enhances the contrast of a digital image by transforming the image histogram in a way that the brightness values become almost uniformly distributed across the entire range of available intensity levels (Fig. 3b1–b2). As an improvement to histogram equilibration (HE) and adaptive histogram equilibration (AHE), in CLAHE an adjustable threshold limits the maximal amount of contrast enhancement in order to avoid the over amplification of noise^46^. CLAHE is also applied for enhancing the contrast of sonographies, CT and MRI data^62,63^.

#### Stripe artefact removal

The occurrence of dark unidirectional stripe patterns is a typical artefact in light sheet microscopy recordings. These stripes are usually generated by small pigmented particles within the sample that obstruct parts of the light sheet. This results in shadows causing reduced fluorescence excitation in stripe shape regions in direction of the light sheet propagation axis (Fig. 3c). To remove these stripe artefacts we developed a software that applies a pie-shaped 2D slope filter in the frequency domain, which selectively attenuates spatial frequency components in the known direction of the stripes (Fig. 3c–e2). Similar filters have been used in other image processing applications before, as e.g. post-processing of satellite images^47^ or geological data sets^64^.

#### Unsharp masking

Final image sharpening (Fig. 3b4) was done using the unsharp-masking filter^65^ implemented in the image processing toolbox of MATLAB (MathWorks, Germany).

#### 3D reconstruction

After imaging and computational post-processing the tumour recordings were 3D-reconstructed using the commercial 3D-reconstruction software AMIRA 6.7 (Thermo Fisher Scientific, Germany).

### Post-UM evaluation of images with standard histological methods

#### Reversal of tissue processing and 4-dots labelling

We labelled the cleared samples immediately after UM-recording and prior to rehydration and paraffin embedding. A “4-dots labelling” was used, where the plane of interest was labelled with DBE-resistant histo-marker (Carl Roth, Germany) at the four sites of the cleared tissue block. Briefly, the sample fixed in a clamp was taken out of the imaging solution, the residues of DBE were removed with tissue paper and the dots were placed at the top and on the side of the specimen. Care was taken to mark this way a plane parallel to the lower side of the specimen holder which is parallel to the imaging plane (Supplementary Fig. S3a–e). After that, the samples were stepwise rehydrated in a descending concentration series of ethanol and finally embedded in paraffin. The 4-dots labelling allowed to correctly position the specimen into the paraffin-chamber, so that the labelling dots remained well-visible after the paraffin-embedding and could serve as orientation marks while slicing with the microtome (Supplementary Fig. S3f–h).

#### H&E staining

Cleared tumour samples were rehydrated by immersing them in descending concentrations of ethanol solutions (100% for 24 h, 80% for 48 h, and 70% for 24 h). After rehydration tissue samples were dehydrated again using an automated system Leica ASP300S (Leica Biosystems, Germany) and subsequently embedded in paraffin. Serial 2-µm-thin sections prepared with a rotary microtome HM355S (Thermo Fisher Scientific) were subjected to histological and immunohistochemical analysis. Serial sections were done in 50 µm steps. Hematoxylin and eosin (H&E) staining was performed on deparaffinized sections with Eosin and Mayer’s Haemalaun (Morphisto, Germany) according to a standard protocol.

DBE was washed out by incubation in absolute ethanol. Re-hydration began with in 2 changes of absolute ethanol, 5 min each, following by 95% ethanol for 2 min and 70% ethanol for 2 min. The samples were then briefly rinsed in distilled water. After that, the samples were stained in Harris’ haematoxylin solution for 8 min and further washed in running tap water for 5 min. The samples were then differentiated in 1% acid alcohol for 30 s and washed under running tap water for 1 min. Afterwards, they were blued in 0.2% ammonia water or saturated lithium carbonate solution for 30 s to 1 min, followed by washing in running tap water for 5 min. Finally, they were rinsed in 95% alcohol, within 10 dips. Counterstain was performed in eosin-phloxine solution for 30 s to 1 min. Then, the samples were dehydrated through 95% alcohol, 2 changes of absolute alcohol, 5 min each, cleared in 2 changes of xylene, 5 min each and finally mounted with xylene-based mounting medium (Thermo Fisher Scientific).

Upon obtaining the histological images, we looked for the best matching optical sections for comparison of the methods and identification of biological tissue structures in UM-recordings (Figs. 5d–h, 6d,e; Supplementary Fig. S3e,h).

#### Immunohistochemistry (IHC)

Cleared tissue samples, which had been recorded by UM were further subjected to IHC staining to demonstrate that our clearing procedure is compatible with subsequent immunostaining. All histological methods were performed in accordance with the institutional guidelines and regulations. Immunohistochemical staining was carried out as previously described^66^.

Immunohistochemistry with progesterone receptor PR, clone 16, PI633C002 (DCS-diagnostics, Germany) antibody was performed on a Benchmark XT—automated stainer (Ventana, Tucson, USA) using the Ultra View DAB Detection Kit (all reagents from Ventana, Tucson, USA). Briefly, the tissue sections were deparaffinized with EZ Prep at 75 °C and 76 °C, heat pretreated in Cell Conditioning 1 (CC1) for antigen retrieval at 76–100 °C and then incubated with the primary antibody diluted in antibody diluent 1:100 for 32 min at 37 °C after inactivation of the endogenous peroxidase using UV-inhibitor for 4 min at 37 °C. The slides were incubated with a HRP Universal Multimer (Ventana, Tucson, USA) for 8 min. Antibody binding was detected using DAB (3,3′-Diaminobenzidine) as chromogen and counterstained with hematoxylin for 8 min with subsequent bluing in bluing reagent for 4 min. Slides were then dehydrated manually by alcohol washes of increasing concentration (70%, 96%, 100%) and xylene and cover-slipped using Pertex mounting medium (Histolab, Sweden) (Fig. 8e,f).

Additionally, immunohistochemistry with cytokeratin pan antibody Z0622 (DAKO, Germany) was performed using a Bond RXm system (Leica, Germany, all reagents from Leica) with a primary antibody dilution of 1:750. Briefly, slides were deparaffinized using deparaffinization solution, pretreated with Enzyme 2 solution for 10 min. Antibody binding was detected with a polymer refine detection kit without post primary reagent and visualized with DAB as a dark brown precipitate. Counterstaining was done with haematoxylin (Fig. 8c,d).

## Supplementary information

Supplementary Information.
