# Supplementary material for: 3D histopathology of human tumours by fast clearing and ultramicroscopy
Source: Sci Rep. 2020 Oct 19;10:17619. doi: 10.1038/s41598-020-71737-w (PMC7572501; doi:10.1038/s41598-020-71737-w)
Supplement: Supplementary file 1 — Supplementary Information. [file 41598_2020_71737_MOESM1_ESM.pdf]

3D histopathology of human tumours by fast clearing and ultramicroscopy

Inna Sabdyusheva Litschauer[1,2] *, Klaus Becker[1,2], Saiedeh Saghafi[1,2], Simone Ballke[4], Christine Bollwein[4], Seyed Meraaj Foroughipour[1,2], Julia Gaugeler[1,2], Massih Foroughipour[1,2], Viktória Schavelová[1,2], Viktória László[3],
Balazs Döme[3], Christine Brostjan[3], Wilko Weichert[4], Hans-Ulrich Dodt[1,2]

[1]Department of Bioelectronics TU Wien, Vienna, Austria.

[2]Center for Brain Research, Medical University of Vienna, Vienna, Austria.

[3]Department of Surgery, Anna Spiegel Center of Translational Research, Medical University of Vienna, Vienna, Austria.

[4]Institute of Pathology, TUM School of Medicine, Technical University of Munich.

Supplementary figure S1.

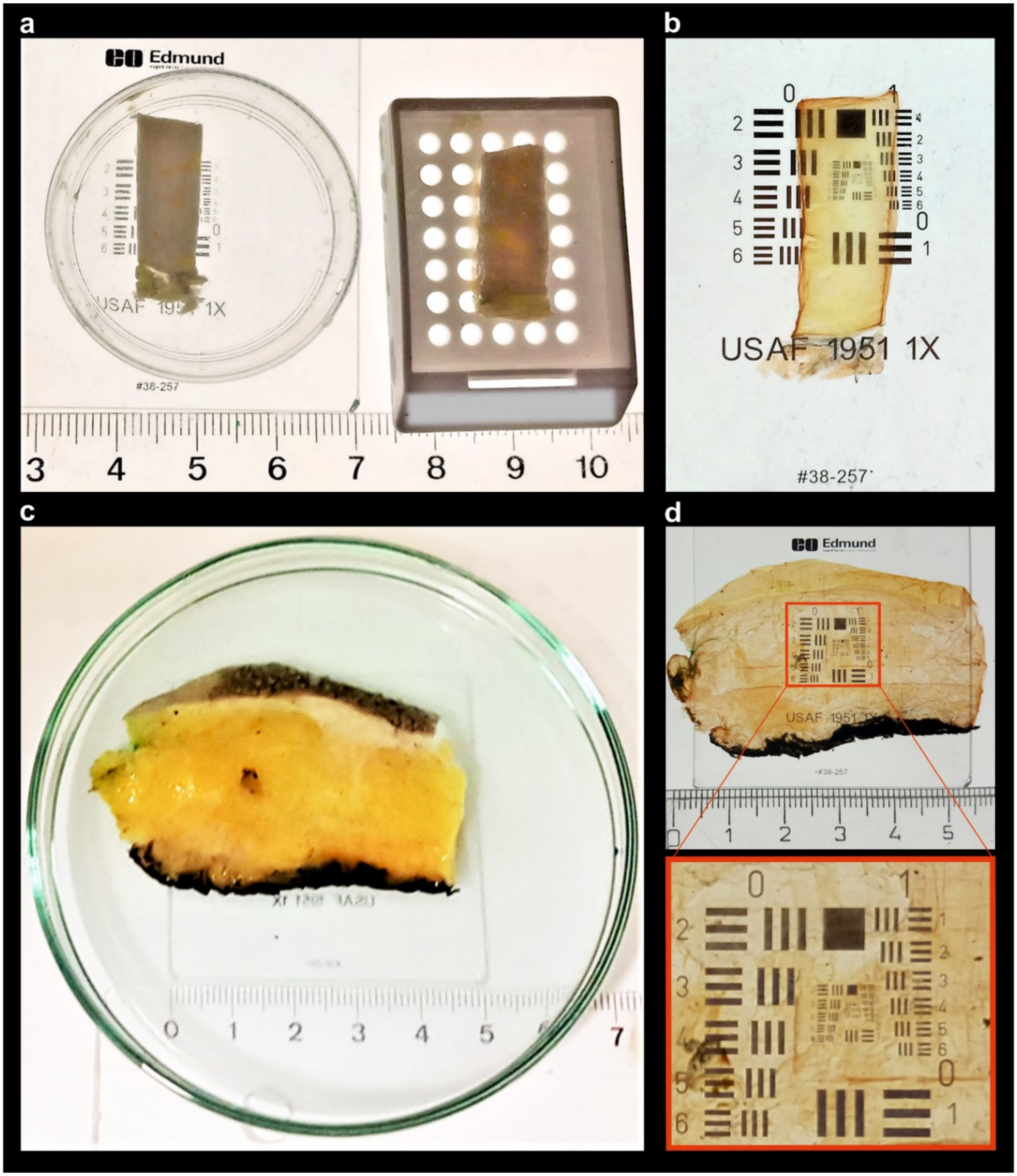

Figure S1. Examples of pathoDISCO clearing technique rendering breast tissue resectates transparent in less than 48 hours. **(a)** Tissue sample in a size of standard histological cassette (30 x 20 x 5 mm) before, and **(b)** after chemical tissue clearing. **(c, d)** pathoDISCO applied to the large tissue sample, in a size of Jumbo-tissue cassette (75 x 52 x 18 mm).

Supplementary figure S2.

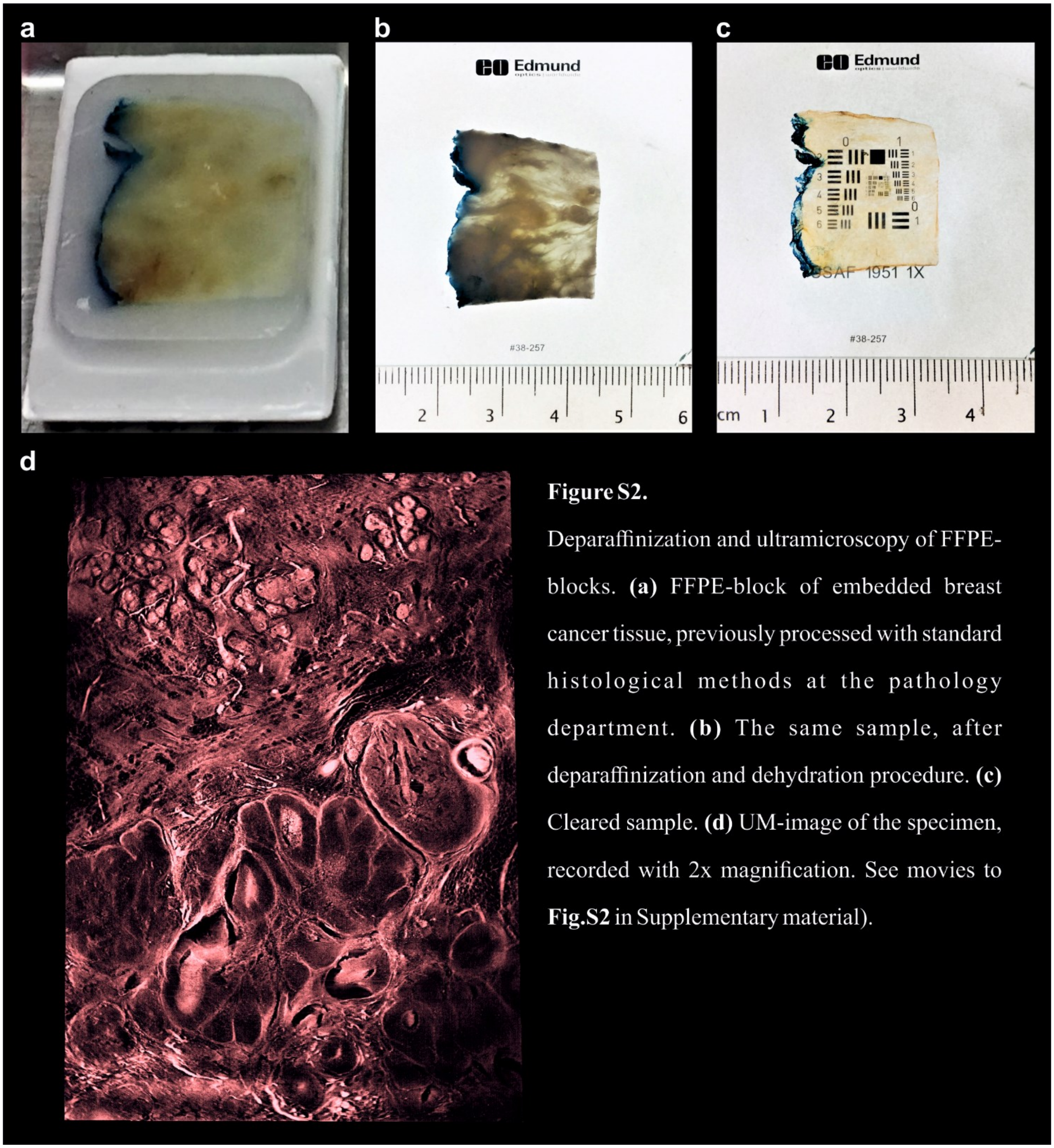

Figure S2.

Deparaffinization and ultramicroscopy of FFPE-blocks. **(a)** FFPE-block of embedded breast cancer tissue, previously processed with standard histological methods at the pathology department. **(b)** The same sample, after deparaffinization and dehydration procedure. **(c)** Cleared sample. **(d)** UM-image of the specimen, recorded with 2x magnification. See movies to **Fig.S2** in Supplementary material).

Supplementary table S1.

Shrinkage comparison*

Volume (LxWxH) of the samples prior tissue clearing, compared to the volume of cleared samples, processed with either pathoDISCO or 3DISCO.
Samples #1-4 (breast cancer tissue resectates) were cut in halves, each half was processed with either method.

Sample ID	Method	Uncleared Volume(in cm^3)	Cleared Volume (in cm^3)	Shrinkage %
1	PathoDISCO	2,016	1,728	25%
	3DISCO	1,68	1,008	40%
2	PathoDISCO	0,567	0,48	16%
	3DISCO	0,63	0,486	23%
3	PathoDISCO	1,215	1,17	4%
	3DISCO	1,62	1,25	25%
4	PathoDISCO	0,96	0,896	7%
	3DISCO	0,864	0,528	39%
5	PathoDISCO	1,65	1,512	9%
6	PathoDISCO	1,872	1,296	31%
7	PathoDISCO	0,882	0,78	11%
8	PathoDISCO	1,053	0,9	14%
* Shrinkage comparison experiments were performed with a limited amount of specimen and serve solely as indication of a mild tissue clearing with pathoDISCO protocol				

Supplementary table S2.

Transparency indices comparison

Measurement of transparency index is described in Materials and methods. Samples #1-4 (breast cancer tissue resectates) were cut in halves, each half was processed with either method.

Sample ID	Method	Transparency Index (TI)**
1	PathoDISCO	14.25
	3DISCO	1.78
2	PathoDISCO	3.56
	3DISCO	14.25
3	PathoDISCO	12.7
	3DISCO	16
4	PathoDISCO	16
	3DISCO	14.25
5D*	PathoDISCO	228.1
6D	PathoDISCO	203.2
7D	PathoDISCO	20.16
8D	PathoDISCO	161.3
*D – deparaffinised samples		
**TI-comparison experiment was performed with a limited amount of samples and serves to demonstrate the high performance of pathoDISCO tissue clearing applied to large specimen		

Supplementary figure S3.

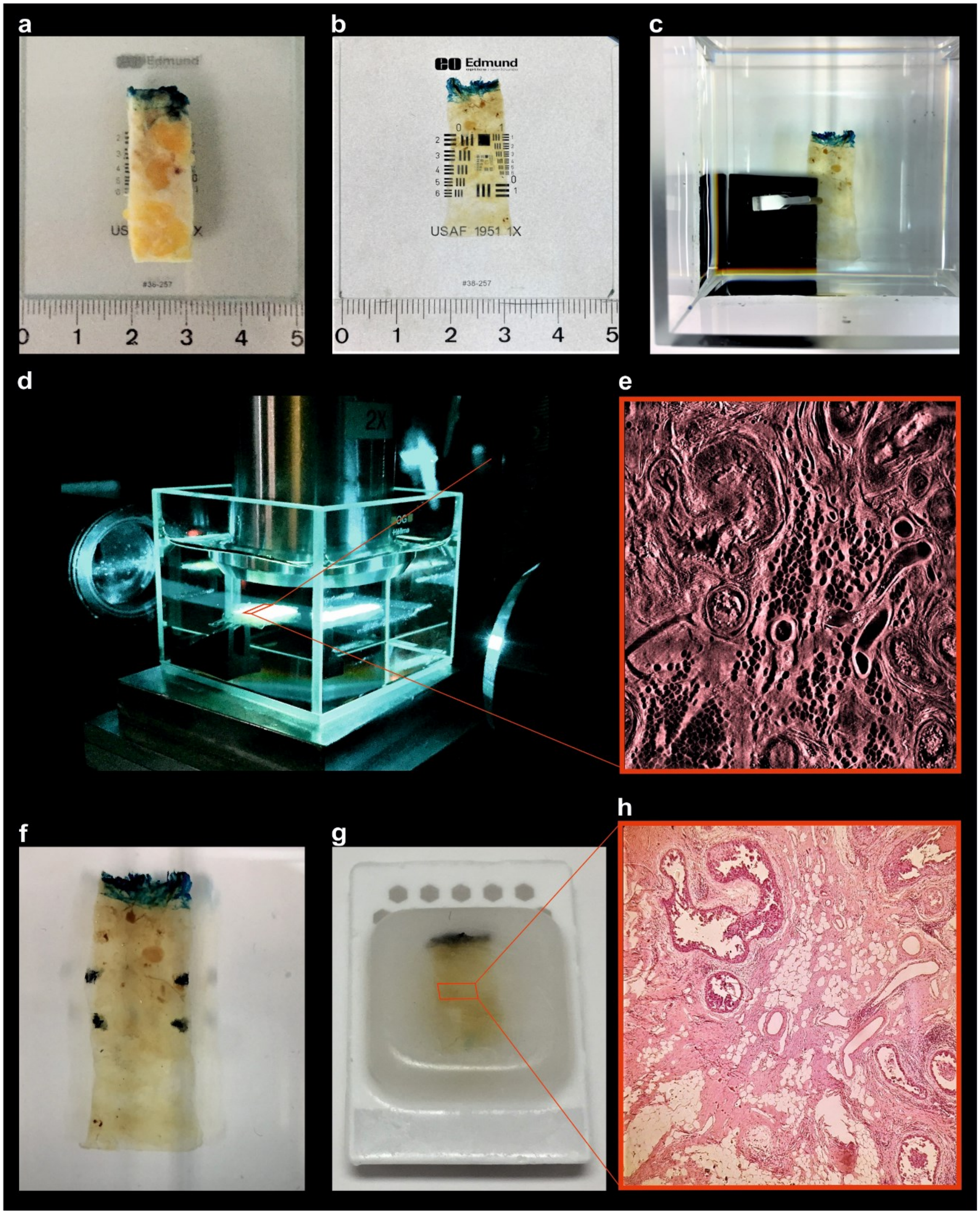

Figure S3. Workflow of 4-dots labelling. **(a)** Uncleared breast tissue specimen. **(b)** Same sample, cleared with pathoDISCO. **(c)** Sample held by a metal clamp, photographed from above, placed in quartz-chamber for **(d)** UM-imaging. **(e)** One of the several thousands of recorded optical planes. **(f)** 4-dots-labelled „plane of interest" immediately after the UM-recording (described in materials and methods). **(g)** Paraffin embedded sample prior microtome-slicing. **(h)** Resulting image of the cut and H&E-stained physical „plane of interest" (note the loss of intraductal cellular mass due to the standard procedure).

Movies

(to watch, please mark the hyperlink and then open it with “open link” in the mouse menu. For better performance, increase the video quality/resolution in menu “Settings”)

All movies represent 3D reconstructions of the tissue samples, shown in Figures. They demonstrate examples of digital slicing (scrolling through the sample), zooming-in and -out and highlighting structures of interest, such as neovascularization.

Figure 4. Application of pathoDISCO for 3D-imaging of human breast neoplasms. Highlighting distinct tissue structures

D1, D 2

2x magnification

https://drive.google.com/open?id=1_rCpZ8ggKuwWnjLAXiSUqfO3klPJ2MES

E1, E2

16x magnification

https://drive.google.com/open?id=1oaWb-eJpzOSEaG5BVrM7DgEaFjrS52TG

Figure 4. F1, F2

16x2 magnification

https://drive.google.com/open?id=1p6YD40qNL8tMTjCBVPIZsSMLnEze26O4

Figure 5. Post-processing of cleared specimen with standard histology methods. 3D reconstruction of the sample, imaged prior standard histology

D, 4x magnification

https://drive.google.com/open?id=1ef5Nd-gcHnzXnOqCaFYP1iXbvN9I6WfT

Figure 6. 3D-imaging of low-grade invasive-lobular adenocarcinoma of breast

2x magnification

https://drive.google.com/open?id=1ZnERImicioENkMaFZOMeaPW-JD-o2cjg

8x magnification

https://drive.google.com/open?id=1Z4P3_2c5usevoKvGDvkJOjerUzfvHknu

Figure 7. Tissue compounds preservation comparison for specimen processed with pathoDISCO and standard histology. 3D reconstructions of samples recorded with UM prior the standard histology

4x magnification

https://drive.google.com/open?id=1345_qKDDYQlsa3PdWrqtytjW8Dibgo2J

16x magnification

https://drive.google.com/open?id=10c6ebfNVymJldgngs_-eVggYwUZ0MINp

Supplementary material videos

Supplementary figure S2. Deparaffinization, clearing and UM -recording of breast-neoplasm tissue, derived from FFPE -blocks

2x magnification

https://drive.google.com/open?id=1MGYxaacaanvg0DOYCtPDH_SRHVXKBknB

16 x magnification

https://drive.google.com/open?id=1KjEi2JL_pqIgvd5JszfhTHfz-suTIG8j
